# The Confluence of Perceiving and Thinking in Consciousness Phenomenology

**DOI:** 10.3389/fpsyg.2017.02313

**Published:** 2018-01-11

**Authors:** Johannes Wagemann

**Affiliations:** Institute for Waldorf Education, Inclusion and Interculturalism, Alanus University of Arts and Social Sciences, Mannheim, Germany

**Keywords:** perception, cognition, history of science/consciousness, phenomenology, first-person observation, perceptual reversal, micro-actions

## Abstract

The processual relation of thinking and perceiving shall be examined from a historical perspective as well as on the basis of methodically conducted first-person observation. Historically, these two psychological aspects of human knowledge and corresponding philosophical positions have predominant alternating phases. At certain historical points, thinking and perceiving tend to converge, while in the interim phases they seem to diverge with an emphasis on one of them. While at the birth of modern science, for instance, these two forms of mental life were deeply interlinked, today they seem to be separated more than ever before – as a number of scientific crises have shown. Turning from the outer to the inner aspect of this issue, a phenomenological view becomes relevant. In terms of the consciousness phenomenology developed by Steiner (1861–1925) and Witzenmann’s (1905–1988) Structure Phenomenology, this article will show how a methodical integration of thinking and perceiving can be carried out on the basis of first-person observation. In the course of a skilled introspective or meditative self-observation the individual’s own mental micro-actions of separating and integrating come into view, jointly constituting what we usually call thinking and perceiving. Consequently, this approach includes a conceptual as well as a perceptual dimension the experimental confluence of which ties in with the methodological core principle of modern natural science. At the same time, making this principle explicit may open the way to a further development of human consciousness and its scientific delineation.

## Introduction

As the two cornerstones of human consciousness, perceiving and thinking have understandably become core issues of modern psychological research. As such they have been extensively explored by means of the methodological framework that has been established in the development of psychology as a scientific discipline during the last 150 years. In the course of this development, many substantial results have been achieved, especially regarding the strong interdependencies of thinking and perceiving as demonstrated in the following discussion. On the one hand, according to the paradigm of “The power of the situation,” it turned out that our thinking and subsequent behavior are significantly influenceable by the things or situations which we perceive. Evidence of this has been presented, for example, in the classical study of Milgram (1963) about destructive obedience as well as in the field of perceptual priming (e.g., Murphy and Zajonc, 1993). Both approaches involve experimental settings in which individuals are confronted with external stimuli prompting them toward specific interpretations or forms of behavior. Other examples are the impact of certain words (Hassin et al., 2005) or the denotation of an experimental game on subsequent behavior (Wall Street Game vs. Community Game, Liberman et al., 2004). On the other hand, with reference to the paradigm of constructivism, it has become common psychological knowledge that what we think about the world or people determines what we actually perceive. Scholars such as Kelly (1955) and Piaget and Inhelder (2000) expounded this principle in terms of developmental psychology or therapy; it has been subsequently substantiated and expanded by application to other phenomena such as social stereotypes (Katz and Braly, 1933) and different aspects of mindfulness (Langer, 2014).

Both directions of effect between thinking and perceiving have been carefully substantiated in numerous empirical studies and can therefore be deemed certain. Moreover, the two modes of psychological interaction functionally fit together and seem to be mutually interdependent; for this reason they can be considered to be based on similar or even the same mental processes. However, at this point one of the methodological core difficulties of modern psychology begins: the inner nature of mental processes cannot be directly observed and measured in the same manner as their external and quantifiable behavioral utterances. Due to the methodological self-restriction of mainstream empirical research to the distanced third-person perspective, fundamental factors of consciousness must be treated, at least in part, as implicit processes (Schacter, 1987; Proctor and Capaldi, 2012). In this view, thinking and perceiving are assumed to be predominantly based on automatic routines running unconsciously or unaware so that they can only be addressed by either unobservable mental constructs or non-mental terms (De Houwer and Moors, 2012). The most common and largely exhausted way of dealing with this methodological deficit is to trace conscious events and processes back to certain forms of brain action (e.g., Dennett, 2001; Cleeremans, 2011). From this angle, perceiving and thinking are supposed to be explained, for example, by various neural measures such as “late amplification of relevant sensory activity” or “long distance cortico-cortical synchronization” (Dehaene and Changeux, 2011, p. 200). In common with most other attempts at explanation, the experiential unity of conscious perceptions as well as of thoughts is reduced to distributed neural activity in the brain (Müsseler and Prinz, 2002).

Today, the doctrine of neuro-reductionism is the predominant official manner of scientifically integrating thinking and perceiving as central aspects of consciousness. But on closer examination, this kind of integration remains speculative in the sense of non-verifiable since there seems to be no experiential bridge between the phenomenal properties of mental and neural processes which could be scientifically examined (Levine, 1983; Noë, 2009). In other words, this theoretical ‘integration’ appears more as a separation because its thinkability implies its non-perceptibility. Looking at it the other way round, the quantitative measures of neuroscience appear, so to speak, as externalized perceptions without any inner coherence on the phenomenal or experiential level. Consequently, their conceptual integration requires a clear distinction between the neuronal as necessary conditions and the experiential as sufficient conditions of consciousness (Nussbaum and Ibrahim, 2012). In this sense, sufficient conditions of thinking and perceiving must comprise certain criteria which can make their genetic and integrative relevance clear on an experiential level. Without questioning the necessity of neural processes for phenomenal consciousness, a more detailed and immanent examination of mental processes should promote our understanding of the other side of the coin: the inner relations of thinking and perceiving as the working mechanisms for consciousness.

In this study, the experiential relations of thinking and perceiving will be investigated in two steps, a preliminary historical and a phenomenological one. While the historical perspective on thinking and perceiving leads to a more theoretical dimension of the topic in the sense of a large-scale picture of philosophical and scientific evolution, the phenomenological view explicitly brings into view the experiential or first-person dimension which so far has been insufficiently considered in science. Furthermore, as shall be shown, the two views are structurally linked to each other and therefore could jointly lead to a comprehensive perspective. Since each of the issues, thinking and perceiving, can be understood in itself in terms of mental action and temporal development, it can be expected that both methodological views, the historical and the phenomenological one, will come together at a certain point of the analysis.

## Historical Development of Thinking and Perceiving

In the following discussion, a few highlights are given from the historical perspective, especially regarding the history of human consciousness and philosophy. In respect to prehistoric times, the available artifacts do not seem to indicate cultures in which perceiving and thinking were experienced as sharply separated forms of mental life, or as different approaches to the world at all. However, since only little is known about prehistoric cultures, this view remains speculative, on the one hand. But, on the other hand, the relatively slow development of cultural knowledge in its initial stages could be an argument that in the early stages of humanity there is still an undivided and world-bound state of mind regarding perceiving and thinking. In this sense, it is probably the absence of written language which could indicate that the state of mind is still holistic and remains in immediate experience and instinctive reaction. Insofar as animal forms of life and consciousness are regarded as human predecessors, the automatic interconnection of perceptual impression and behavioral expression obviously refers to an earlier stage of development. In turn, the historic emergence of written language can be taken as a first expression of how humanity becomes conscious of a growing mental gap between immediately lived experience – normally focused on the perception of the outer world – on the one hand, and the inner process of reflecting one’s experience – thinking about something – on the other. The act of writing something which has been thought or experienced saves these events from passing away with the current of time and makes it possible for them to become increasingly distinct. However, prehistoric cave paintings, for example, could be interpreted as a preliminary step in this direction, even though they still remain in a pictorial, pre-linguistic, and therefore probably dreamlike, pre-rational form of consciousness. Nevertheless, in addition to creation myths that are orally handed down in all cultures, this already shows the beginning of the separation of subject and object. Later on, in ancient Indian philosophy and then at the dawn of modern Western consciousness in pre-Socratic philosophy, the subject–object split became more and more solidified whereas there are only few indications of a further differentiation between perceiving and thinking as distinct forms of approaching the world (Windelband, 1957). We can regard Parmenides’s (520/515–460/455 B.C.) and Empedocles’s (495–430 B.C.) speculations in epistemology and philosophy of nature as examples of the rather uniform experience of sensorial and cognitive functions (Capelle, 1963). However, the fact that possible epistemic relations between human individuals and the surrounding world are the object of intellectual deliberation already indicates an incipient divergence of perceiving and thinking in these philosophers. Starting with this implicit emancipation from the immediately perceived world as well as from religious associations, this new, autonomous thought in philosophy irresistibly became a necessary condition for the further disentangling of perceiving and thinking.

**FIGURE 1:**
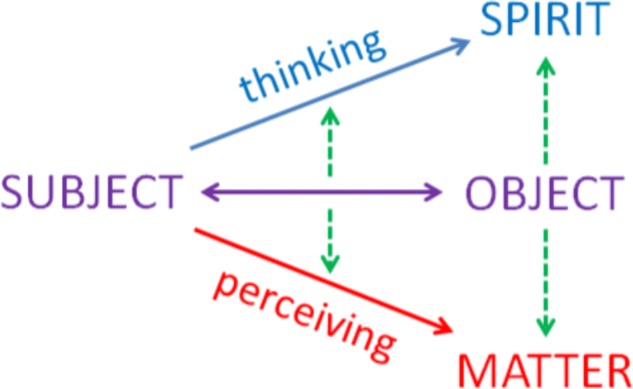
Differentiation processes in the history of consciousness. The basic process is the increasing separation of subject (human being) and object (world). Closely interwoven with this is the differentiation of the cognition object into a spiritual aspect and a material aspect and the successive realization of equivalent forms of knowing–thinking and perceiving.

It may be that a sharp distinction of different forms of knowledge at first explicitly occurred in the history of consciousness with Democritus (460/459–371 B.C.) and Plato (428/427–348/347 B.C.). In the course of more exact thought about the sources of knowledge the specific characteristics of thinking and perceiving emerged and began to move away from each other. Since, they are oriented toward different aspects of the world such as the multitude of impressions conveyed by the sense organs or the unity and coherence brought about by thinking they diverged into different, even conflicting world views – as clearly seen in Plato’s idealism and Democritus’ early form of materialism. Plato’s way of obtaining real and sure knowledge was the ascent of thought toward the realm of ideas, the anamnesis in which the soul rediscovers its forgotten access to divine coherence and lawfulness (Platon, 2002). However, although he dismisses the epistemological value of sensory perception, Plato associates the aim of the philosopher’s development initially launched by thought with a non-sensory form of experience or metaphysical perception of the ideas (in German: *Ideenschau*). In contrast, Democritus preferred an explication of all existence in spatial and material terms. His atomism assumed smallest particles that were supposed to constitute everything, be it material or mental. These atoms or particles appear as theoretical abstractions from single objects perceived through our bodily senses (Windelband, 1957). Therefore, at that time, the split between different forms of human self-experience gradually came to awareness – be it as a material body in a material world or as an immaterial soul in a spiritual world – and therefore the abyss between perceiving and thinking flew open (**Figure 1**).

This diverging development and its consequences can be further traced throughout the history of philosophy and science. In the Scholastic philosophy of the middle ages, this divergence can be illustrated by the question whether ideas have to be considered to be real, spiritual entities – accessible to thought – or whether they are human constructions articulated as words or names for things which first have to be sensually perceived in the outer world (Klima, 2016). Moreover, it was characteristic of Scholastic philosophy that this question was only discussed in theoretical terms within the methodological frame of syllogistic inference, excluding empirical investigation and evidence. Later on, in the Renaissance, the discovery of perspective as a means of visual representation opened the possibility of distinguishing between aspects of spatial relation and contextual meaning (Gebser, 2010). Previously, in early Christian and medieval painting, the ‘status perspective’ (in German: Bedeutungsperspektive) used the size of represented characters only to depict their hierarchic status or their degree of sanctity and not as an expression of perceived spatial depth. With the era of the Enlightenment and the beginning of the modern era, this development culminated in the philosophical differentiation of rationalism and empiricism. As emphasized by René Descartes, rationalism favors self-referential, logical and consistent thought because this alone can provide a sound basis for integrating all the continuously changing and uncertain perceptions of our senses (Descartes, 1641/1959). According to him, thought is the most important tool of both self-knowledge and world knowledge and is inherently independent of outer perception. In turn, as proclaimed by John Locke, empiricism favors comprehensive and precise sensory experience, perceiving or measurement since it is only in these that access to sure knowledge can be found: “[…] when does a man begin to have any ideas? I think the true answer is: when he first has some sensation. Since there appear not to be any ideas in the mind before the senses have conveyed any in […]” (Locke, 1690/1836, p. 60).

Interestingly, despite these opposing stances, both epistemological paradigms already converged in a certain sense in the birth of modern natural science at the beginning of the 17th century. They did not, of course, converge as a restoration of the former state of mind of prehistoric times. Rather, the opening of modern science was an innovative step toward a state of mind which was the most advanced one at that time – simply because of the methodological integration of perceptive and cognitive skills at the highest level. Without a clear insight into the laws of mathematics together with profound experimental observation, the enormous success of natural science would not have happened. Galilei spoke about the book of nature written in mathematical language (Galilei, 1623/1960). In order to read this book, we have to look precisely into nature but we also have to be able to decipher its cryptic, mathematical character. The first is needed in order to perceive, the second in order to think. Or vice versa: without thinking, we have no idea, no hypothesis about what to see when looking into nature. In the adequate methodical combination of theoretical thought and experimental observation and measurement lies the secret of scientific success. But it has to be pointed out that this new methodology contained (and to this day still contains) the danger of a fatal imbalance.

**FIGURE 2:**
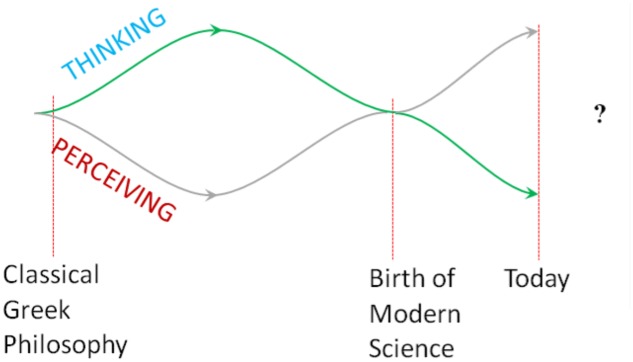
Divergence and confluence of thinking and perceiving.

The ‘dark side’ of modern science relates to another dictum, ascribed to Galilei, condensing the self-confident and extensive claims of the quantitative research attitude (Kleinert, 2009): what can be measured, must be measured, what cannot be measured, must be made measurable. While this is a powerful program for investigating physical phenomena, it leads to serious questions when simply transferred to other disciplines such as biology, psychology, and sociology. For the natural sciences such as physics and chemistry, a materialistic ontology seems to be quite suitable – although not necessary. But what happens in the attempt to measure even those phenomena that obviously contain an experiential and hence immaterial aspect? Phenomena such as life and conscious experience are simply reduced to their outer, behavioral and thus measurable expression. As already mentioned in the introduction, when such a form of scientific ‘imperialism’ is pursued, the inner or qualitative dimension of phenomena is marginalized so that in the end it seems to be without effect or completely non-existent. In historical comparison, this is a complete reversal of the Scholastic one-sidedness of treating all problems in the rigid intellectual scheme of logical argumentation without experimental validation. Since Galileo’s and Newton’s days, modern science is increasingly at risk of slipping into the other one-sidedness of reducing all questions and phenomena to physically measurable data. In psychology, as a human science, this initially led to the division of rational and empirical orientations in research in the 19th century and later on to the dominance of the latter. However, it should be noted that the aspiring branch of empirical psychology was strongly aligned with the methodological paradigms of natural science and therefore dismissed all aspects of introspective observation. Here, the investigations of the Würzburg school of introspection could serve as an example which is more likely to show that psychological interests generally shifted toward a behaviorist method than that introspective research is inherently inadequate (Danziger, 1980; Weger and Wagemann, 2015a). In the course of this shift a paradox becomes relevant in modern psychology: on the one hand, as a researcher, the individual has enormously increased his power of methodological control and therefore emancipated himself from religious patronizing and metaphysical or superstitious attitudes. On the other hand, as an issue of research, the individual tends to become a measurable and hence controllable object without much self-awareness or self-efficacy, especially when it comes to establishing the psychological mechanisms. Although in the end, it is actually the same human being that once appears as an active subject and then as a passive object of science, considerable efforts are normally made to keep these aspects neatly separated. In the course of this development, the researcher increasingly becomes alienated from himself as a conscious being and this, in consequence, has fostered the belief in materialism. What can be perceived or measured – outer objects – and what can be thought – inner representations or constructions – these two aspects have again drifted apart from each other (**Figure 2**).

In conclusion, we can draw an interim inference containing the following three aspects. Firstly, the brief excursion into the history of consciousness, philosophy and science has shown certain dynamics which can be termed as a successive separation and integration of thinking and perceiving, or as a divergence and confluence (Wagemann, 2016a,b). This dynamic in the history of consciousness can be considered as a hypothetical pattern of development. In certain historical stages, these styles or paradigms of knowing^1^ and associated world views seem to periodically diverge and converge. Secondly, especially during the periods of divergence, a particular cultural emphasis on thinking or perceiving appears to become present which leads to a predominance of idealistic or materialistic world views, of rationalism or empiricism. As can be seen here, in this first period of about 2000 years the predominance of neo-Platonism, Patristics and Scholastic philosophy in the context of the growing influence of Christianity speaks for a more idealistically tuned phase without much interest in rigorous empirical observation. However, at the same time and almost independently, the empirical conditions for modern science silently emerged in the course of these centuries, for example the invention of technical instruments and measurement and the training in practical experimental skills, for instance in the alchemistic laboratories of the middle ages. And then, at the birth of modern natural science in the coincidence of mathematical thinking and quantitative observation, the turning point toward the unstoppable victory march of materialism was reached. Even though this development was temporally overlapped by German idealism, this merely seemed to spur on the liberation of science from all metaphysical ballast. – Altogether, the development of consciousness throughout history appears to proceed in a periodic movement of thinking and perceiving. But, thirdly, this oscillation does not just appear as an eternal recurrence of the same but as a progressing evolution involving well-known movements as well as entirely new qualities. The rise and fall of periodically emerging fashions of thinking and perceiving such as idealism and materialism is strongly aligned to the human striving for emancipation and individualization. This striving seems to be a continuously transforming dimension of human consciousness with quite new, ever-expanding and unpredictable forms of expression and effect. To sum up, we can distinguish (1) the historical basic pattern – separating and integrating – of thinking and perceiving, (2) the predominance of thinking or perceiving in certain phases, and (3) the strong tendency of striving for emancipation and individualization as a guiding principle throughout all phases.

This can serve as a working hypothesis and as a background against which we can briefly look at the current situation. As already mentioned, we seem to live in a period in which thinking and perceiving are quite separated from each other. Regarding the psychological topic of vision, for example, perceptual and conceptual forms of knowledge are neatly distinguished for reasons of processing speed and the difference between particularity in perception and generality in conceptual thinking (Gregory, 1997; Dretske, 2000). Besides such more discipline-specific aspects, the main indication of this separation can be seen in the fundamental crisis that modern science and philosophy are still unable to give a consensual answer to the most challenging questions such as “What is being?,” “What is consciousness?” Despite enormous efforts to promote neurosciences in the last decades there is no solution to the hard problem of consciousness in sight, i.e., there is no striking and consistent idea as to how and why conscious phenomena such as thinking and perceiving are correlated to brain action (Chalmers, 1995). Nevertheless, in the current *Human Brain Project*, for example, the attempt is made to technically simulate brain processes on larger scales in the assumption that this would already provide a better understanding of human consciousness (e.g., Hahne et al., 2015; Dehaene et al., 2016). In this way, ironically, the mainstream of cognitive science and materialistic neuro-philosophy proclaims a certain unity of perceiving and thinking – simply in the sense that both are equally affected by brain processes.

We already argued above that a ‘neuro-centric’ attempt at ‘integration’ is quite ill-conceived with regard to two important aspects: (1) Methodologically, it has a high price due to the impossibility of experientially accessing the processual origins of thinking and perceiving in ongoing conscious experience. (2) Logically, this account could not properly differentiate between somatopsychic and psychosomatic directions of effect, which should be understood as the necessary and sufficient conditions of consciousness processes. – In addition to these arguments, the following discussion will briefly provide further reasoning. (3) The significant steps in the differentiating evolution of thinking and perceiving, as the historical analysis has shown, clearly took place after the termination of the neuroanatomical evolution of the human brain about some 10,000 years ago (Eccles, 1989). Attributing the development only to neural reorganization and subsequently increased connectivity remains hypothetical (because brain tissue cannot be studied by paleontology) and also would not surmount the categorical gap of explanation (Wagemann, 2010). (4) If the sober distinction between scientific observation – perception – and interpretation – thought – is taken seriously, the character of neural processing does not give any hint of the generation of phenomenal consciousness and, even less, of the integration of thinking and perceiving. Rather, neural signal processing shows a character of modal de-qualification and structural decomposition in relation to the properties of the contents of consciousness (Witzenmann, 1983; von Foerster, 1998; Laurence and Margolis, 2001). Furthermore, no brain region or neural algorithm could be found which would substantiate any integrative effect (Wagemann, 2010, 2011). (5) For the brain, in comparison with other physical organs such as the eye or the stomach, no integrative status can be postulated because none of these organs is functionally self-referential. The eye cannot totally see itself (even not in a mirror) and the stomach cannot digest itself (this would be pathological), so why should the brain establish an integrative self-reference of the whole person? Rather, it would be useful to speak of functional self-exclusion regarding all physical organs without exception (Wagemann, 2010). In other terms, this argument has become known as the mereological fallacy confusing one part (brain activity) with the whole (conscious human being) (Bennett and Hacker, 2003).

With a view to these arguments indicating the functionally insufficient and divisive character of neural processes and to the recent historical development of science, especially psychology, leading to a methodological dissociation of thinking and perceiving, it has been shown that new ways toward integration should be sought. In the following discussion, one prospect pointing in this direction will be demonstrated in the context of Structure Phenomenology as a specific form of consciousness phenomenology comprising methodological aspects as well as an introspective pilot study.

## The View of Consciousness Phenomenology

Instead of strictly adhering to the third-person paradigm articulating itself in distanced measurement and formal argumentation – which can be rated as artificially externalized and distanced forms of perceiving and thinking – approaches such as the phenomenology of perception (Merleau-Ponty, 1962/1965) or neurophenomenology (Varela, 1996; Lutz and Thompson, 2003) and other kinds of phenomenological (e.g., Albertazzi, 2013) and introspective research (e.g., Petitmengin and Bitbol, 2009; Weger and Wagemann, 2015a) take the first- and second-person perspective into consideration anew. After the preliminary failure of introspective accounts at the beginning of the 20th century the approaches in question work on different methodological aspects of introspection in order to gain new insights into psychological phenomena. Without distracting from the importance of standard psychological research, such investigations can show that first- and second-person accounts are not only valuable additions to the former but can also bring fundamental structures to light that might otherwise remain hidden (Petitmengin, 2007; Weger and Wagemann, 2015b; Weger et al., 2016, 2017).

Against this background, a distinct approach of consciousness phenomenology called Structure Phenomenology shall be presented here and applied to the question of the inner relation of thinking and perceiving. This approach has already been touched in some of our former studies mentioned above and shall now be shifted to the foreground. This seems to be justified because this form of consciousness phenomenology includes certain methodological aspects that make it possible to approach the core processes of consciousness more closely than do more common accounts (Wagemann, 2010). The crucial point, as shall be shown in the following discussion, lies in a clear and systematically replicable relation of consciously experienced mental action and structural components of reality. Structure Phenomenology was established by Witzenmann (1905–1988) in the eighties of the last century as a further development of Steiner’s (1861–1925) epistemology and consciousness phenomenology as well as Goethe’s (1749–1832) method of natural research (Witzenmann, 1983). Witzenmann also assimilated some influences from Hegel, Husserl, and Heidegger, especially in his terminology, but with some significant shifts of method and meaning. Therefore, Witzenmann’s approach must not be confused with similarly named concepts such as Structural Phenomenology which are more substantially rooted in the Husserlian tradition (Rombach, 1980; Brown, 2005). The key aspect of Steiner’s and also Witzenmann’s concept was to transform Goethe’s method of natural research into an epistemologically clarified method of consciousness research (Steiner, 1924/2003; Witzenmann, 1987). That means to (1) identify specific forms of mental action which regularly occur in the observation of outer natural phenomena (plants, colors, etc.), (2) detach them from their self-imposed restriction to sensorial stimuli, (3) turn them toward the consciousness process itself, and (4) observe ongoing mental processes within the constitution of common conscious phenomena, e.g., within thinking and perceiving, in a methodologically enhanced state of consciousness. This approach locates phenomenology precisely between the exercise, observation and description of mental acts in concrete situations, on the one hand, and the endeavor to search for their invariant processual structure, on the other. Therefore, to a certain extent, it stands between the more pragmatic forms of descriptive (Giorgi, 2009) or experimental (Ihde, 1986, 2012) phenomenology and the turn to transcendental idealism of the late Husserl (Zahavi, 2009). Regarding the extensive debates in cognitive science and philosophy of mind, Structure Phenomenology indeed refers to some central aspects discussed as will be shown, but, however, stands out in terms of its fine-grained empirical method and its processual conception of mental events. Methodologically, Structure Phenomenology has a certain proximity to Petitmengin’s research regarding “performative coherence” as a criterion of validity immanent to consciousness (Bitbol and Petitmengin, 2013, p. 270). This criterion could also be applied to certain mental “micro-operations” (p. 276) or “micro-gestures” (Petitmengin and Bitbol, 2009, p. 380) which are normally executed unconsciously but can be brought into consciousness. In this sense, performative criteria regarding certain forms of mental action could denote what was mentioned above as the sufficient conditions of consciousness. Not surprisingly, with reference to the historical excursus, and in line with Steiner’s and Witzenmann’s findings, the basic structure of these mental gestures can be identified as a continually alternating dynamic of separation and integration. Already for Goethe, separation and integration were the essential forces or actions constituting existence in the world as well as human knowledge (Goethe, 1977, p. 32). They serve, so to speak, as a ‘dynamic bridge’ between nature on the one hand and the researcher on the other.

**FIGURE 3:**
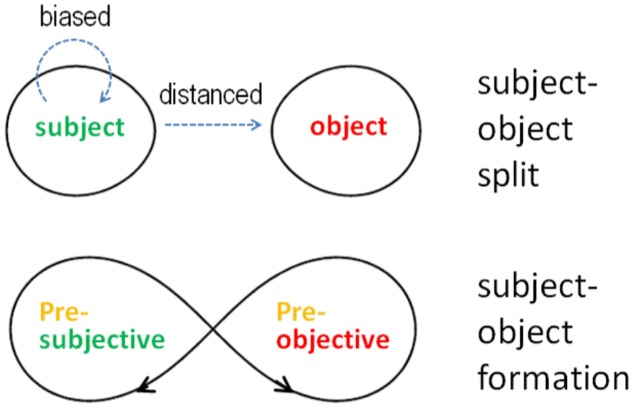
Research attitudes. The common research attitude of modern science pre-supposes the *subject–object split*, whereas the meditative approach of Structure Phenomenology gains experiential access to the *subject–object formation.*

As Witzenmann showed, the transformation of Goethe’s method can also be applied to the gap between subject and object in modern scientific consciousness (Witzenmann, 1983, 1987). To the extent that he remains in the subject–object split, the researcher is only able to collect and reflect objectively distanced or subjectively interwoven and therefore biased data. While the latter is rightly discarded as non-scientific, the former complies with the paradigm of standard scientific research. Neither of these attitudes, however, can lead to the original formation of subject and object since they presuppose the results of this process. Equally, the mental attitude of the subject–object split does not offer any way to understand thinking and perceiving in a constitutive sense. Here, Structure Phenomenology offers an epistemological conception and method to observe states that are normally pre-subjective and pre-objective in the ongoing genesis of individual consciousness (**Figure 3**). These processual states can be accessed by becoming increasingly aware of the separating and integrating forms of mental action that we are constantly performing in our everyday thinking and perceiving. The crucial point here is to trace Goethe’s epistemic principles of separation and integration back to the pre-reflective origin of mental action in which object and subject do not yet fully exist but are in the process of emerging. This includes, inter alia, that the generation of subject and object also goes through phases of non-conceptual representation or reference what has been hypothetically discussed in philosophical and cognitive science debates (e.g., Evans, 1982; Heck, 2000; Pylyshyn, 2009). However, contrary to Marr (1980) and Raftopoulos and Müller (2006) and as a central empirical finding of Structure Phenomenology, the formation of subject and object cannot be solely ascribed to subpersonal brain action because its relevant starting points and sufficient conditions cannot be found there. Rather, they show up in the interaction between the pre-objective (necessary) conditions conveyed by the neural system and the pre-subjective (sufficient) conditions performed by the individual mental agent. Here, the former conditions phenomenally appearing as non-conceptual challenges and the latter conditions providing conceptual qualities together allow for the formation of the subject–object relation. Otherwise, as indicated above in the fourth argument against neuro-centrism, the non-conceptual aspect of perception could be associated with the functional character of neural decomposition.

The structural elucidation of mental processes from the first-person perspective, of course, requires a specific training concerning the degree of awareness in these processes. In other words, although they normally run pre-reflective or subconsciously – like an undercover agent – the actual genesis of our everyday consciousness can itself be made conscious to us – which would amount to the unmasking of the implicitly operative agent who we are ourselves in the proper sense. Precisely because the relevant mechanisms of action in thinking and perceiving processes are forms of mental self-efficacy, they cannot be accessed via external measurement, randomization, and blinding but rather as methodically guided introspective self-recognition. In this context, Ned Block’s distinction between ‘phenomenal consciousness’ and ‘access consciousness’ (Block, 1995) appear to be useful insofar as there obviously exist certain aspects of mental self-efficacy (A-conscious) which are not necessarily P-conscious at the same time. However, pursuing the idea that perception without attention may be impossible (Rock, 1997), more recent studies confirm the trainable ability of P-consciousness of gaining access to aspects of cognitive und perceptual processes formerly regarded to be exclusively A-conscious (Slagter et al., 2007; Petitmengin et al., 2013). Hence, an apodictic gap between these types of consciousness, even for the example of blindsight patients, does not seem to be expedient for a methodical advanced exploration of the first-person perspective.

**FIGURE 4:**
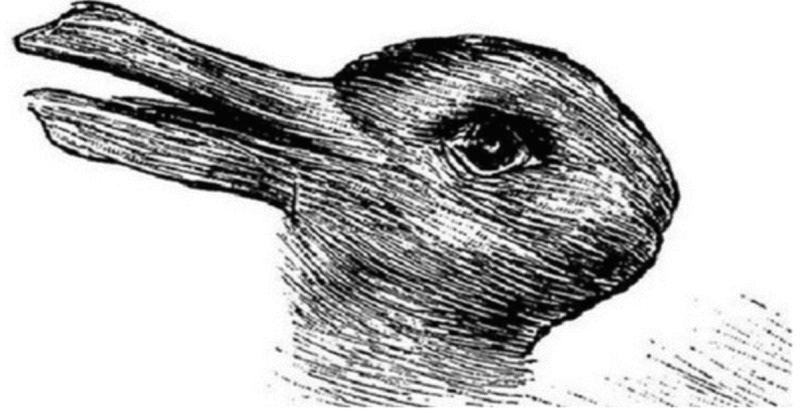
Duck or rabbit? (Jastrow, 1899, p. 312).

Let me provide two examples to illustrate what this means. Paradoxically, the best starting point for this is precisely a lack of clarity. As in normal science, in Structure Phenomenology it also begins with the moment of uncertainty or ambiguity. Only what is unclear is worth investigation and can thus lead to a research question. A quite artificial variant of this principle are picture puzzles (see **Figure 4**). Depending on viewing habits, in this picture you may see a duck or a rabbit. With some conscious effort and a little practice we are able to switch between the two possible variants as we please (Liebert and Burk, 1985). So what is ultimately depicted in this image? In terms of a definitive result it cannot be clearly said. Most likely, we might assume that it is neither a duck nor a rabbit but simply the “or.” To this extent, this seems to be nothing spectacular, but in dealing more intensively with this “or” phenomenon, we may realize that the appearance of the whole world in our consciousness works in the same way as a picture puzzle. An important difference, however, is the fact that there are not only two but rather infinite possibilities of seeing the world, even the easiest thing, in totally different aspects through one and the same pair of eyes. On the other hand, as everyday experience shows, there is no arbitrariness regarding the fit of conceptual possibilities and perceptual stimuli.

In Don Ihde’s framework of an Experimental Phenomenology, these levels of insight are distinguished as “literal-minded” (only one variant is seen) and “polymorphic-minded” (different variants could be volitionally seen) (Ihde, 2012). However, since a polymorphic-minded attitude is still focused on different possible results of seeing, a further ascent to a ‘processual-minded’ attitude regarding the generation of each individual instance of seeing is still pending. If we carefully observe the volatile transition point in the perceptual change in which the former variant is no longer valid and a new variant has not yet come into view we would only notice a short moment of irritation. This moment seem to refer to what Fred Dretske has denoted as ‘sense perception’ in contrary to ‘conceptual perception’ (Dretske, 1990, p. 132). Normally, we don’t care about this stage of pure, non-conceptual sense perception, because we simply ignore all perceptual irritations which cannot be immediately and routinely categorized. Hence, they do not exist for us, at least for the moment. But when we maintain our awareness of such unclear situations of momentary non-existence, they lead us to an epistemic crisis, although crisis does not necessarily mean an explicit and manifest psychological event, which would have to be fully conscious to the individual.^2^ Rather, crisis means ‘decision’: in view of the incoherent sensorial impression, we have to decide what can be seen. In the easiest case, as in the example, there are two possibilities, two variants of how to decide. And these possibilities of vision are nothing other than certain thought contents which have to be produced by our own thinking activity. Deciding what to see means to previously think a potentially corresponding thought content. What we cannot think we are equally unable to see. And, vice versa, what we are actually thinking forms what we are going to see. In becoming aware of our actual thinking, these phenomena do not remain theoretical constructs of implicit processes but may become accessible in an experiential sense. Occasions for a pertinent mental training can be found in artificial picture puzzles such as the one discussed above, but also in our everyday perception.

At this point, let me present the second example: one morning in the winter, I was driving on the highway in my car. The morning was quite foggy and I was entering a long bridge over a river valley when the whole world beyond the bridge (which normally comes in sight at this moment) was veiled by an impervious, white curtain. Suddenly, while driving on the bridge, I became frightened because directly in front of me a dense, dark smoke ascended above the road. Immediately, I had the impression that a serious accident had occurred and instinctively reduced speed. However, in the next moment, I felt quite relieved, because I realized that there was actually no smoke and consequently no accident. It was just the slowly rising fog releasing a view of the dark silhouette of the forest on the other side of the bridge. The ‘smoke’ I saw was simply the negative image of the vanishing fog. Since everything was ok now, I tried to return to the ‘smoke’-condition of vision. Surprisingly, it worked, and it worked so well that the unpleasant feeling I previously had about the smoke also returned for a second. Then, prudently, I stopped my phenomenological experiment in order to avoid any actual accident.

Later on, I analyzed this strange experience, asking myself what it was that I was actually doing. Obviously, the dark smoke I initially saw was an illusion. But certainly an illusion which can be deliberately invoked, as became apparent in my experiment. Hence, as a first finding, the limitations of volitional influence on perceptual reversals as alluded to by Merleau-Ponty (1962/1965, p. 307/342) seem to be less constraining than presumed. So this phenomenon cannot be reduced to what is normally called a perceptual illusion due either to bodily mediated sense (phenomenology of the body) or to subpersonal construction ascribed to the brain (neuro-reductionism). I initially had to produce, more or less consciously through my thinking action, what I finally perceived. And this thinking action had to come into resonance with certain thought contents or concepts that are fully coherent in themselves – although they did not necessarily lead to stable perceptions. Therefore, we can speak of *thinking within perceiving*, on the one hand. On the other hand, in this experience, I realized the possibility of introspectively perceiving my own ongoing thinking and perceiving actions which are normally focused on outer events and therefore blind to themselves. By embracing the structure-phenomenological key concepts of ‘concept’ and ‘percept,’ ‘thinking action’ and ‘thought content,’ among others, which can open the doors of processual mental observation, it seems to be possible to turn attention inward by instantaneously looking outward. In other words, the attention in thinking and perceiving processes could be split into two parts, a heteronomous and an autonomous one (Witzenmann, 1983), and therefore we can also speak of a specific *perceiving within thinking*.

**FIGURE 5:**
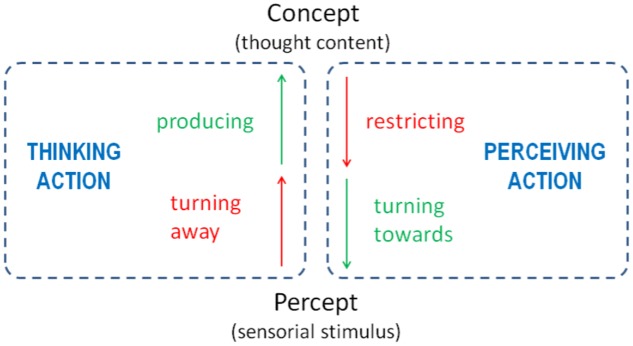
Complementary structure of perceiving and thinking. In the structure-phenomenological inquiry of a perceptual reversal four micro-gestures of mental action can be distinguished. They can be generalized as forms of mentally separating (turning away, restricting) and integrating (producing, turning toward).

A closer investigation of the phases we undergo in such experiences leads to the following conclusion in line with the structure-phenomenological conception. The quite surprising option to deliberately see the world as false – the dark smoke which I could repeatedly evoke – shows that I first had to leave the proper interpretation of the percept – the rising fog before the dark background. In order to change the view, it is necessary to discard the previously seen content in favor of another meaning structure; each content only becomes available by producing a thought content. In other words, the first two steps include a separation and an integration. The separation refers to the former perceptual content, the integration to an expected content which is not yet perceived. It has to be actualized by thinking activity which means an inner, integrative and holistic gesture. But because thinking of dark smoke does not amount to actually seeing dark smoke, the actualized thought content has to be oriented and focused toward the unclear perceptual field. This focusing accompanied with a restriction of the universal conceptual range results in a search movement approaching and scanning the visual field in order to find appropriate anchorage points. And in the end, if successful, the dark smoke is actually perceived – even if there is none – with a brief, habitual certainty. To sum up, the last two steps include both a separation and an integration. The separation refers to the universal horizon of meaning of the thought content in the sense of its restriction to an individual case of perception. And in restricting all thinkable expressions of dark smoke to only one, the thought content receives the form of being merged with the perceptual stimulus. In this way, the leading thought content becomes individualized and will therefore be able to build an integrated whole together with the stimulus. Only now, as a consequence of the preceding mental events (as sufficient conditions), does the stimulus that was initially passively perceived (as a necessary condition) reach the state of active, conscious existence. Otherwise, I would not have seen it as real for a moment. Only then does it become true in the sense of the German word “Wahrnehmung,” which literally means “taking for true.” According to Steiner and Witzenmann, these phases could be summarized as the phenomenal action of thinking and perceiving; and in the course of Structure Phenomenology, thinking becomes perceivable (in the experience of introspective observation) and perceiving becomes thinkable (in recognizing the coherent dynamic structure as analyzed above) (**Figure 5**).

Regarding the above remarks on non-conceptual representation, we are now able to embed this notion in the described basic structure of separating and integrating mental actions. Exactly, at the transition point between turning toward to the stimulus and turning away from it the former stable perception disappears with open eyes before a new thought content has been grasped. In this moment, we feel slightly irritated because we are exposed to the raw, non-conceptual experience of the stimulus. However, this processual stage denoted above as an epistemic crisis, has not to be confused with W. James’ “pure experience” since the latter contains separated as well as integrated phenomenal aspects (James, 1912/2003; Wagemann, 2010). Rather, this transition point of ‘non-conceptual representation’ – representing nothing that could be coherently addressed as something – indicates the final, degenerated stage of thoughtful (conceptual) observation and, again, stimulates a further act of thinking. Due to its phenomenal quality, it could be interpreted as a consciousness-related effect of the neural activity offering decomposition (separation) as well as the opportunity of recomposition (integration) of our mental world. The latter, however, in its consciousness-related effectivity, cannot be delegated to the brain, but rather has to be mentally performed and optionally observed in wakeful mental action.

## Conclusion

To sum up, using the structure-phenomenological method, we can analyze our thinking in perceiving or perceived thinking in perception as a continuous oscillation between mental actions or micro-gestures whose forms we can denote as separating and integrating. Although this is a mental structure as indicated by Steiner and Witzenmann and illustrated here by individual examples, it does not remain merely subjective. The crucial point here is that this first-person research approach does not stick to individual sensations, which could be suspected of being externally influenceable and internally deceitful. Rather, it works with distinct forms of mental action serving as phenopractical tools or, psychologically spoken, as independent variables which could be deliberately controlled by the introspecting researcher. The voluntary and aware use of these mental variables leads to reproducible conditions and intersubjectively verifiable mental states and structures in the sense of performative coherence (Bitbol and Petitmengin, 2013, see above). Therefore, this processual structure can be taken as a second working hypothesis for further investigation in first-person consciousness research. Compared with older or newer hypothetical constructs of information processing (e.g., Gregory, 1997; Tononi et al., 2016) this approach to the relation of thinking and perceiving makes distinct progress in terms of conceptual clarity and direct observability. Further consistency could be achieved by referencing back to the first part of this study. While the first hypothesis that I presented refers to the historical development of human consciousness, the second hypothesis refers to the ongoing genesis of consciousness in our minds. These two perspectives, which at first were discussed separately from each other, now seem to show the same structure. While the historical perspective shows the mental movements of separation and integration on a larger temporal scale, of which the people of past epochs remained largely unaware, the view of introspective or meditative phenomenology may unveil this structure today in our own conscious experience and experienced process of consciousness. In other words: what formerly operated as an implicit process tends to become explicit 1 day. Thus, the challenge is to clear the fog in this field in order to investigate further the hypothesis of an isomorphic structure of consciousness processes on different temporal scales.

In this way, we can establish a new synthesis of thinking and perceiving in modern consciousness phenomenology as a consequence of the previous historic development and of our own systematically trained experience. As shown, human performances of consciousness historically diverged into thinking and perceiving and passed through different phases of predominance, with an alternating bias for each form of knowledge. With the emergence of modern science, the former inwardness of a period emphasizing pure thought was overcome and compensated by the strong intention to observe and measure the outer things themselves instead of fruitlessly discussing them. But very soon, the ingenious combination of theoretical thought and quantitative data collection adopted the other one-sidedness of a predomination which emphasized the outer, perceptual or measurable world. Here, as indicated above, the previous development of psychology may serve as an instructive example. Consequently, the essential benefit of modern empirical science goes beyond all the great and highly specialized discoveries made by physicists, chemists, biologists, and psychologists. Rather, it can be seen in the inner methodical relation of thinking and perceiving that has become apparent through the past centuries, although initially limited to outer, material fields of enquiry. However, it would be tragically mistaken to rest on this. As the philosopher Jean Gebser said, all great ideas which have become leading principles for whole epochs tend to evolve to one-sided and deficient forms of expression (Gebser, 2010). So, why not dig out the buried core of science, purify it from its materialistic contamination and refine it to yield a new, differentiated and integrative science of consciousness?

## Author Contributions

The author confirms being the sole contributor of this work and approved it for publication.

## Conflict of Interest Statement

The author declares that the research was conducted in the absence of any commercial or financial relationships that could be construed as a potential conflict of interest.

## Footnotes

1 This could be considered as a variation and further extension of Ludwik Fleck’s well-known concept of ‘thought styles’ and ‘thought collectives’ into wider dimensions of the evolution of consciousness (Fleck, 1980).

2 Here, references to Dewey (1909); Waldenfels (2006), and Oevermann (2008) can be made since all of them, in their specific ways, emphasize the importance of a critical starting point in their epistemologies. For instance: “[..] the origin of thinking is some perplexity, confusion, or doubt” Dewey (1909, p. 12).
